# No causal association between the volume of strenuous exercise and coronary atherosclerosis: a two-sample Mendelian randomization study

**DOI:** 10.3389/fcvm.2024.1344764

**Published:** 2024-04-25

**Authors:** Zijie Xiao, Guolin Huang, Guanhong Li, Huihui Wang, Xiaoyu Zheng, Yongchun Li, Fengying Gong, Ying Lv, Jingjun Li

**Affiliations:** ^1^ Nanfang Hospital, Southern Medical University, Guangzhou, Guangdong, China; ^2^ School of Traditional Chinese Medicine, Southern Medical University, Guangzhou, Guangdong, China; ^3^ The Second School of Clinic Medicine, Guangzhou University of Traditional Chinese Medicine, Guangzhou, Guangdong, China

**Keywords:** Mendelian randomization, the volume of strenuous exercise, coronary atherosclerosis, cardiac rehabilitation, genome-wide association study, high-intensity interval training

## Abstract

**Objective:**

Several observational studies have shown that high-volume and high-intensity exercise training increases the prevalence and severity of coronary atherosclerosis, but the causal effect still remains uncertain. This study aims to explore the causal relationship between the volume of strenuous exercise (SE) and coronary atherosclerosis (CA) using the Mendelian randomization (MR) method.

**Method:**

The exposure factors were two basic parameters of the volume of strenuous exercise (duration and frequency of strenuous exercise), the outcome factor was coronary atherosclerosis, and the relevant genetic loci were extracted from the summary data of the genome-wide association study (GWAS) as the instrumental variables, and MR analyses were performed using the inverse variance weighting (IVW) method, the weighted median method, and the MR-egger method. Sensitivity analyses were performed using heterogeneity analysis, pleiotropy analysis, and the “leave-one-out” method. The original results were tested using other coronary atherosclerosis data sets.

**Result:**

IVW results showed no causal association between duration of strenuous exercise (DOSE) [OR = 0.9937, 95% CI (0.9847, 1.0028), *P* = 0.1757] and frequency of strenuous exercise (FOSE) in the last 4 weeks [OR = 0.9930, 95% CI (0.9808, 1.0054), *P* = 0.2660] and coronary atherosclerosis. All of the above results were validated with other coronary atherosclerosis data sets.

**Conclusion:**

The present study supports that the causal association of duration and frequency of SE with CA was not found, and provides valuable insights into the choice of scientific and correct volume of SE to cardiac rehabilitation (CR).

## 1 Introduction

Cardiovascular disease is one of the most threatening diseases to people's lives and health worldwide. About 19.05 million people die each year from cardiovascular disease, including up to 9.14 million who die from coronary heart disease(CHD) (1). Prevention and treatment of CHD is a pressing issue that must be addressed. The American Heart Association (2) created Life's Essential 8 to maintain a healthy heart, including active physical activity, maintaining a healthy weight, knowing your cholesterol, not smoking or using smokeless tobacco, eating heart-healthy foods, controlling blood pressure, controlling cholesterol, controlling blood glucose, and getting enough sleep. Among them, exercise is the most effective strategy to reduce the risk of cardiovascular events in patients with CHD. Cardiac rehabilitation (CR), based on exercise training, is now recognized as an important component of comprehensive CHD treatment, effectively reducing the risk of cardiovascular events, all-cause mortality, and hospitalization (3). Numerous studies have demonstrated that the benefits of exercise training show gradual augmentation in tandem with increased exercise intensity and volume. Engaging in strenuous exercise (SE) for 2 h per week can yield health benefits comparable to those derived from moderate-intensity exercise for 4 h per week (4). SE is also more effective than moderate-intensity exercise in reducing cardiovascular events and improving CHD risk factors than moderate-intensity exercise (5, 6). In addition, top-level athletes typically have a longer life expectancy than the general population and a lower risk of death from cardiovascular disease (7). The dose-response relationship between exercise and CHD has attracted increasing attention, but to date, the understanding of the relationship between exercise intensity and exercise volume and the risk of CHD has not been unified. Several recent observational studies have pointed out that high-volume and high-intensity exercise training will actually increase the prevalence and severity of coronary atherosclerosis (CA) (8, 9). Subjects who exercise more (mainly SE) are also observed to have more coronary atherosclerotic plaque. However, another study showed that the proportion of SE in the total exercise dose did not significantly affect the progression of plaques (10). It is worth noting that all existing evidence is based on observational studies. Traditional observational studies, although capable of exploring the etiology of the disease, are inevitably interfered with by measurement errors, confounding factors, and reverse causation. Therefore, clarifying the causal relationship between the volume of SE and CA could help provide recommendations and guidance for exercise training in CR.

Mendelian randomization (MR) is a data analysis technique for assessing etiological inferences in epidemiological studies, which uses genetic variation in exposure factors as an instrumental variable to assess the causal relationship between exposure factors and outcomes. In contrast to traditional observational studies, MR avoids confounding and reverse causation, making the results obtained from MR more plausible (11). In this study, we used a two-sample MR analysis to evaluate the causal relationship between two basic parameters of the volume of SE (duration and frequency of strenuous exercise) and CA.

## 2 Methods

### 2.1 Data sources

In this study, we performed a two-sample MR analysis using genetic variance from the genome-wide association study (GWAS) and adopted single nucleotide polymorphisms (SNPs) as instrumental variables to assess the causal association between components of the volume of SE and CA. The volume of exercise is determined by duration, frequency, and intensity. However, when it comes to clinical practice, the total volume of exercise can be expressed as time spent in SE (12). As a result, as basic parameters of the volume of SE, duration of strenuous exercise (DOSE) and frequency of strenuous exercise (FOSE) in the last 4 weeks were used as the exposures, and CA was used as the outcome. We obtained summary data from the IEU Open GWAS database (https://gwas.mrcieu.ac.uk/datasets/). Instrumental variables of DOSE and FOSE were obtained from a study by Ben Elsworth of the UK Biobank database, which investigated strenuous sports in the participant population through a questionnaire (Supplementary Table S1). The GWAS data on DOSE contained 46,993 samples and 9,851,867 SNPs, and FOSE contained 46,945 samples and 9,851,867 SNPs. Genetic instruments for CA were obtained from a study of the Neale lab, which contained 361,194 samples and 13,586,589 SNPs. To further testify to the results of these exposures and CA, we additionally adopted the GWAS data from the FinnGen consortium, which contained 211,203 samples and 16,380,447 SNPs. To minimize statistical differences that may result from population stratification, participants in this study were selected from the European population (13). The details are shown in (Table 1).

**Table 1:** Data sources.

Trait	Year	Sample size	Sex	Number of SNPs	Ancestry	GWAS ID
Exposure						
DOSE	2018	46,993	Males and females	9,851,867	European	ukb-b-1046
FOSE	2018	46,945	Males and females	9,851,867	European	ukb-b-9991
Outcome						
CA	2018	361,194	Males and females	13,586,589	European	ukb-d-I9_CORATHER
CA*	2021	211,203	Males and females	16,380,447	European	finn-b-I9_CORATHER

DOSE, duration of strenuous exercise; FOSE, frequency of strenuous exercise; CA, coronary atherosclerosis; CA*, validation dataset for coronary atherosclerosis.

### 2.2 Selection of SNPs

**Figure 1:**
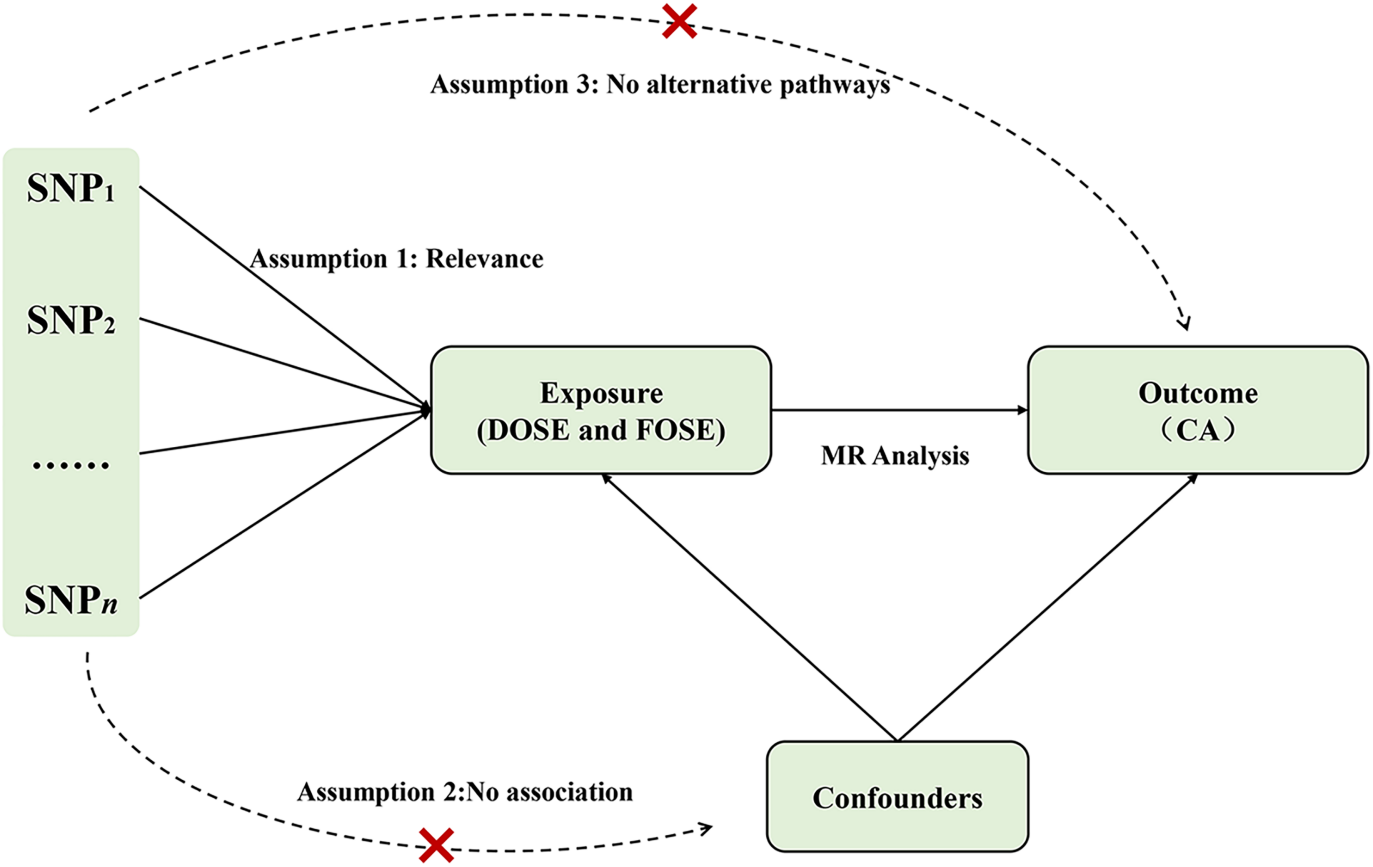
Assumptions of the Mendelian randomization (MR) analysis for exposure (DOSE and FOSE) and the risk of CA. The “×” means that genetic variants are not associated with confounders or cannot be directly involved in outcome but via the exposure pathway. SNP, single-nucleotide polymorphism; DOSE, duration of strenuous exercise; FOSE, frequency of strenuous exercise; CA, coronary atherosclerosis.

In order to obtain unbiased results in MR analysis, the three main assumptions of MR analysis were necessary in selecting instrumental variables: (1) the instrumental variables are closely related to the exposures; (2) the instrumental variables are independent from confounding; and (3) the instrumental variables have an effect on the outcome only through the influence of the exposures (14). The selection of instrumental variables is shown in (Figure 1).

The selection of instrumental variables was based on stringent screening criteria, thus genome-wide significant (*P* < 5 × 10−8) with linkage disequilibrium (*r*^2^ < 0.001, kilobase distance, kb = 10,000) SNPs were selected as genetic instruments for the MR analysis. Considering that the DOSE related-SNPs (*P* < 5 × 10−8) and the FOSE related-SNPs (*P* < 5 × 10−8) rarely exist, we selected genome-wide significant (*P* < 5 × 10−6) SNPs. Nine related SNPs were identified for DOSE and FOSE respectively. The MR-Pleiotropy RESidual Sum and Outlier method indicates no existence of outliers (Table 2) and we found no confounding factors of each SNP in the Phenoscanner database. Next, the palindromic SNPs were removed, and finally, 7 SNPs for DOSE and 8 SNPs for FOSE were adopted in the following MR analysis. *F*-statistic was used to determine the strength of each selected instrumental variable. Usually, *F*-statistic > 10 indicates the absence of weak instrument bias (15). After calculation, the *F*-statistic of each SNP was greater than 10, which showed a strong correlation between instrumental variables and exposures without weak instrumental variable bias (Supplementary Table S2). As for the validation dataset CA, the instrumental variables that were finally included in the MR analysis were the same as those in the original dataset.

**Table 2:** Heterogeneity, pleiotropy test and MRPRESSO of MR studies.

Exposure	Outcome	MR-Egger (*P* value)	IVW (*P* value)	MR-Egger intercept (*P* value)	MR-PRESSO	
					MR-PRESSO global test	Outlier
DOSE	CA	0.3466	0.4412	0.6658	0.6256	0
FOSE	CA	0.0375	0.0548	0.6755	0.0954	0
DOSE	CA*	0.1144	0.1661	0.7144	0.1044	0
FOSE	CA*	0.6479	0.6742	0.4443	0.3942	0

DOSE, duration of strenuous exercise; FOSE, frequency of strenuous exercise; CA, coronary atherosclerosis. CA*, validation dataset for coronary atherosclerosis. MR-PRESSO, MR-Pleiotropy RESidual Sum and Outlier.

### 2.3 MR analysis

The MR analysis was realized by the “TwoSampleMR” and “MRPRESSO” packages of R software (version 4.3.1). We employed three commonly used methods to perform the MR analysis, for instance, the fixed-effect IVW method calculated the weighted average of the effect values of all the chosen SNPs under the assumption that all SNPs are valid instrumental variables. As a result, the IVW method has the greatest statistical efficacy and is the most commonly used analytical method in MR analysis. The weighted median estimator method can be able to carry out an effective MR analysis under the assumption that at least 50% of the chosen SNPs are valid instrumental variables and obtains unbiased dependent effect values, but the statistical efficacy is lower than that of the IVW method. The MR-egger method can provide a valid assessment of causal associations even when all SNPs are invalid instruments. In addition, it also provides evaluation of the pleiotropy among instrumental variables with the intercept term. In this study, the IVW method was used as the most dominant of the three analytic methods, with the weighted median method and the MR-egger method as supplements (16, 17). *P*-values < 0.05 were considered as the threshold for statistical significance among all the above statistical analyses, and it was suggestive of a relatively stable causal association when the direction of the effect value *β* was the same in all three methods. Odds ratios (OR) and 95% confidence intervals (CI) were used as evaluation indicators.

### 2.4 Sensitivity analysis

Several sensitivity analyses were performed to evaluate the robustness of the results. First, the heterogeneity of all SNPs included in the MR analysis was assessed by Cochran's *Q* test (18). With a *P*-value > 0.05 indicating the absence of heterogeneity, the fixed-effects IVW method was considered as the primary method. On the contrary, if a *P*-value < 0.05 suggests the presence of heterogeneity, as a result, a random-effects IVW method should be used (19). Second, we also performed the pleiotropy test to assess the presence of horizontal pleiotropy in multiple instrumental variables. *P* < 0.05 suggests that the outcome is affected by factors other than the exposure, which suggests that the results of the MR analyses are not reliable, and the outlier SNPs should be identified and excluded from MR analysis. In contrast, *P* > 0.05 suggests that there is no evidence of horizontal pleiotropy. Third, the principle of “leave-one-out” is to eliminate each SNP in turn and then perform MR analysis on the remaining SNPs to observe whether the effect value changes significantly, so we can see whether each SNP has a substantial effect on the causal relationship between exposure and outcome.

## 3 Results

### 3.1 MR analysis

**Figure 2:**
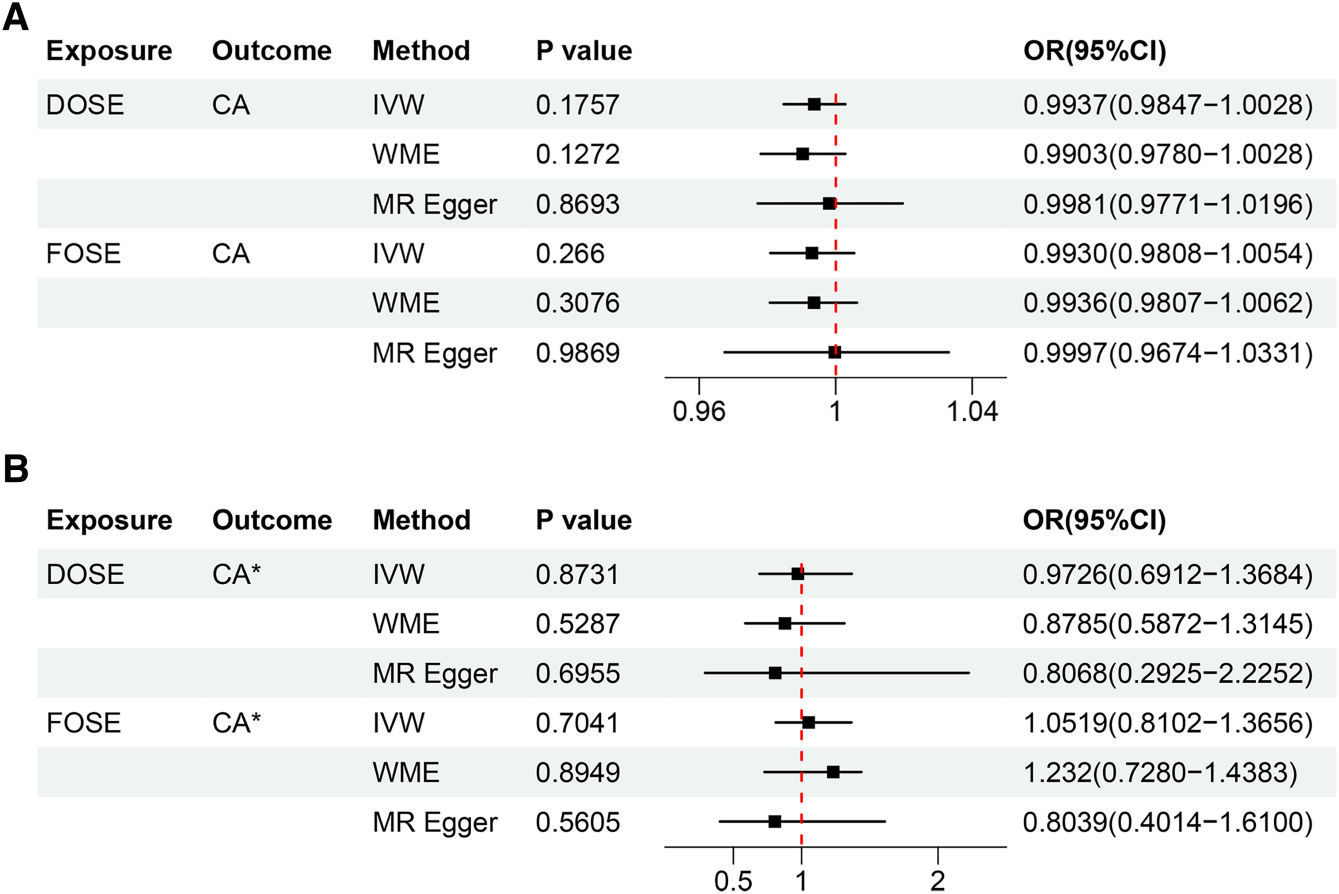
(**A**) Estimation of the causal relationship between DOSE/FOSE and CA using different MR methods. (**B**) Estimation of the causal relationship between DOSE/FOSE and CA* using different MR methods. DOSE, duration of strenuous exercise; FOSE, frequency of strenuous exercise; IVW, inverse variance weighted; WME, weighted median; OR, odds radio; CI, confidence interval; CA, coronary atherosclerosis. CA*, validation dataset for coronary atherosclerosis.

**Figure 3:**
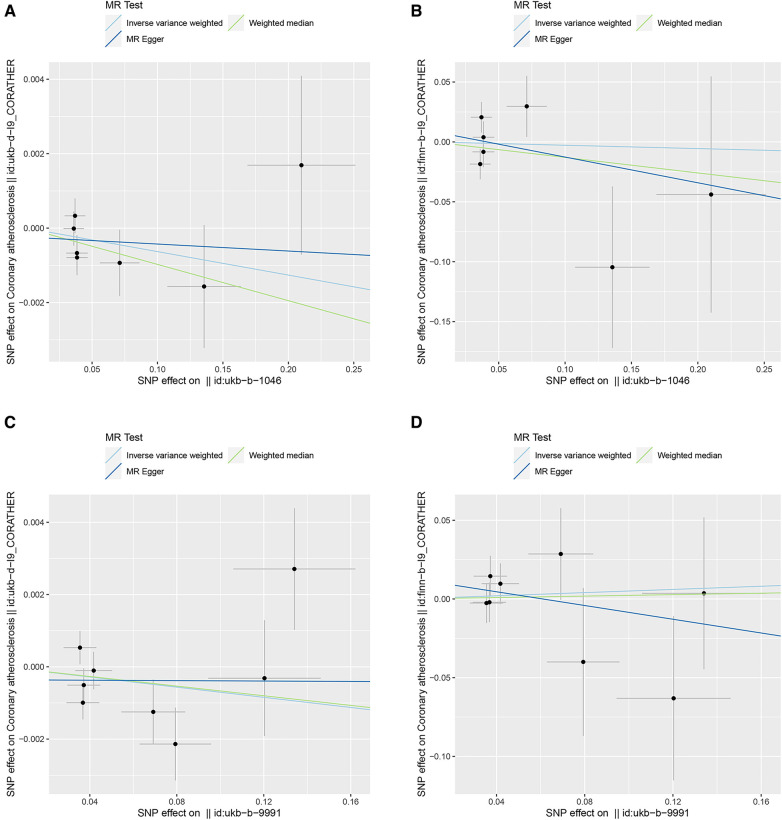
Scatter plots of genetic correlations of DOSE/FOSE and CA/CA*. The *x*-axes represent the SNP effect on exposures; The *y*-axes represent the SNP effect on outcomes. Each line represents a different MR method. (**A**) Scatter plot of genetic correlations of DOSE with CA. (**B**) Scatter plot of genetic correlations of DOSE with CA*. (**C**) Scatter plot of genetic correlations of FOSE with CA. (**D**) Scatter plot of genetic correlations of FOSE with CA*. DOSE, duration of strenuous exercise; FOSE, frequency of strenuous exercise; IVW: inverse variance weighted; WME: weighted median; OR: odds radio; CI, confidence interval; CA, coronary atherosclerosis. CA*, validation dataset for coronary atherosclerosis.

The results of the IVW method showed that DOSE (OR = 0.9937, 95% CI = 0.9847–1.0028, *P* = 0.1757) and FOSE (OR = 0.9930, 95% CI = 0.9808–1.0054, *P* = 0.2660) were not causally associated with CA, with both of their *P* values were greater than 0.05. What's more, with the *P* values of both the weighted median method and the MR-egger method being greater than 0.05, the causal estimation results tended to be in line with those of the IVW method, which indicated the robustness of the results (Figure 2A). Similar trends were observed in DOSE (OR = 0.9726, 95% CI = 0.6912–1.3684, *P* = 0.8731) and FOSE (OR = 1.0519, 95% CI = 0.8102–1.3656, *P* = 0.7041) with validation dataset CA, which further proved that there was no causal association between them (Figure 2B). The scatter plot illustrated the estimated impact of SNPs on DOSE/ FOSE and CA/validation dataset CA (Figure 3).

### 3.2 Sensitivity analysis

**Figure 4:**
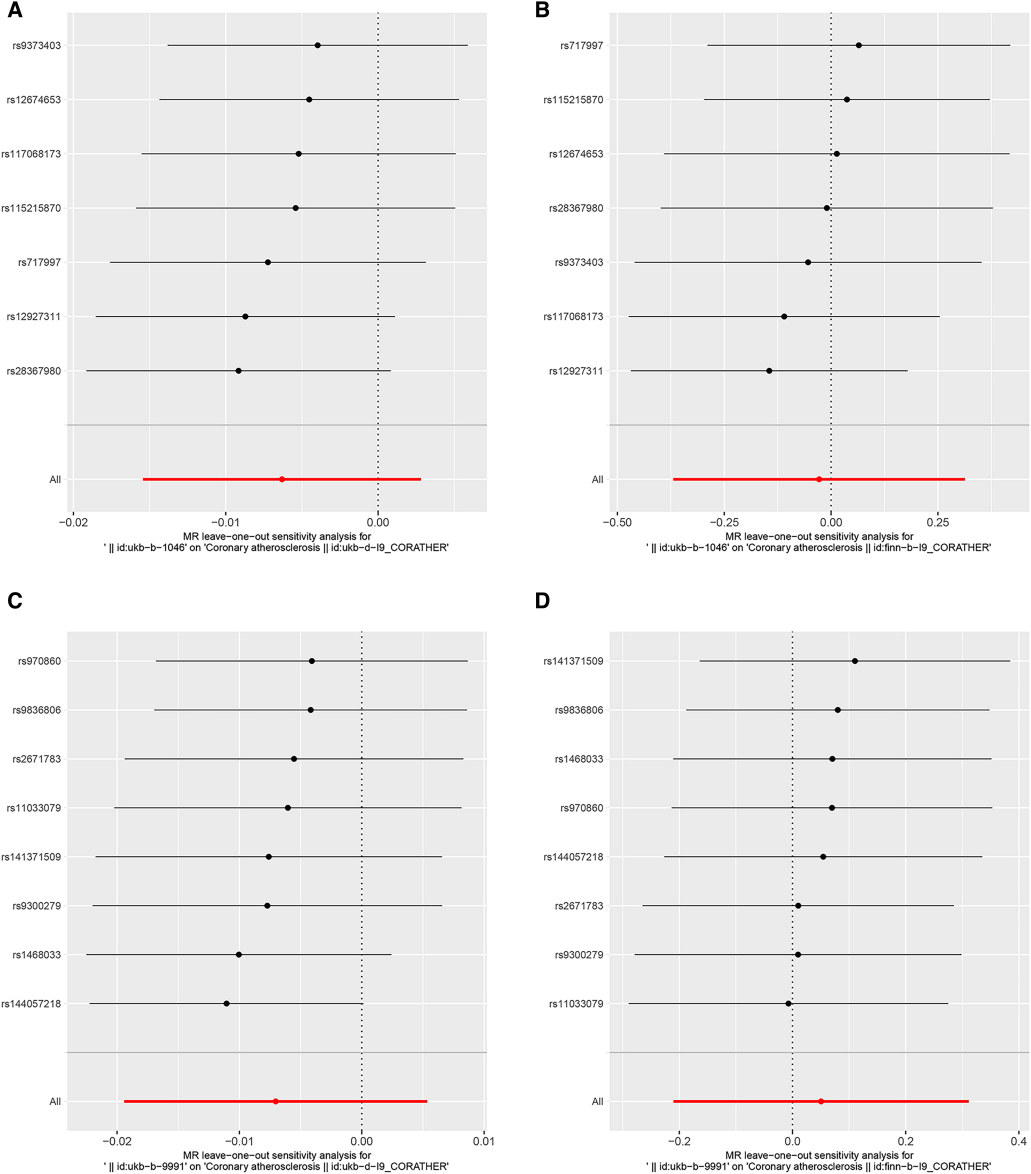
Leave-one-out plots to visualize the causal effects of DOSE/FOSE on CA/CA*. The *x*-axes represent associations of genetically predicted DOSE and FOSE with CA and CA*. The red line at the bottom represents 95% CI of the associations of genetically predicted DOSE and FOSE with CA and CA* with all SNPS added together. (**A**) Leave-one-out analysis for DOSE with CA. (**B**) Leave-one-out analysis for DOSE with CA*. (**C**) Leave-one-out analysis for FOSE with CA. (**D**) Leave-one-out analysis for FOSE with CA*. DOSE, duration of strenuous exercise; FOSE, frequency of strenuous exercise; CA, coronary atherosclerosis. CA*, validation dataset for coronary atherosclerosis.

The heterogeneity test suggested that there was no heterogeneity in the instrumental variables of DOSE. Some heterogeneity may exist in the instrumental variables of FOSE, as the MR-Egger *P*-value of heterogeneity is 0.0375, and the IVW *P*-value is 0.0548, approaching significance (Table 2). Therefore, we used the random-effects IVW method in evaluating the causal relationship between FOSE and CA. In addition, the pleiotropy test indicated that none of the analyses showed the presence of pleiotropy (Table 2). The leave-one-out method for the MR analysis of DOSE and FOSE with CA, indicated that no SNPs were detected that had a large effect on the overall results, which suggested that the causality has a certain degree of stability (Figures 4A,C). As for validation dataset CA, no heterogeneity or pleiotropy existed (Table 2) and the leave-one-out method of the MR analysis (Figures 4B,D) indicated that the exclusion of any single SNP significantly affected the whole results as well, which meant that the causal relationship derived from the MR analysis of the validation dataset were robust.

## 4 Discusstion

In this study, we used Mendelian randomization to explore the causal relationship between two basic parameters of the volume of SE (DOSE and FOSE) and CA. In order to exclude the influence of other exposure factors on the results, sensitivity analysis was performed to verify that the MR Analysis results had certain stability. The results of the MR Analysis and the validation dataset together support the conclusion that there was no causal correlation between the volume of SE and CA. Our results are consistent with the findings of the Aengevaeren study (8), which investigated the progression of CA in 289 middle-aged and older male athletes and assessed its association with exercise volume over 6.3 years of follow-up, showing no significant association with the volume of SE. Coronary atherosclerotic calcification is a specific marker of CA, which reflects the burden of coronary atherosclerotic plaque and is closely related to the risk of atherosclerotic cardiovascular disease (20). The coronary atherosclerotic calcification score plays an important role in the stratification and intensive treatment of identified atherosclerotic risk factors (21). Malik (22) conducted a 30-month observational study and found that as the weekly exercise time increased, the coronary atherosclerotic calcification score of the subjects in the SE group only slightly decreased, but not significantly (*P* = 0.42). However, some researchers also hold different opinions on this view. Bosscher et al. compared the prevalence of coronary artery plaque in 176 control groups, 191 late-onset endurance athletes, and 191 lifelong endurance athletes through computer tomography. The athletes who participated in high-intensity endurance sports all their lives showed more plaque numbers and a higher overall burden of CA (9). In another cross-sectional study of master athletes, it was found that the duration of SE increased the risk of chronic atherosclerosis (23). In these studies, the mechanisms by which SE promotes atherosclerotic plaque progression may involve hypertension, mechanical stress, hemodynamic changes, changes in vitamins, minerals, and hormones, inflammation, or other confounding factors. For example, Rubies (24) simulated the long-term SE training period in humans through a rat model. The results showed that long-term SE may promote adverse vascular remodeling through the processes mediated by the renin-angiotensin-aldosterone system, miR-212/132, miR-146b, and MMP9. However, the progression of plaques caused by SE does not mean an increased risk of adverse cardiac outcomes. In contrast, exercise training will promote the increase of coronary artery diameter, coronary vasodilation capacity, and coronary blood flow reserve, and coronary atherosclerotic plaque associated with increased exercise volume may also be more stable. These may further reduce the risk of cardiovascular events (25). Although SE could promote the expression of inflammation in the short term, in the long run, SE could also promote the anti-inflammatory phenotype transformation of lymphocytes and monocytes, thus promoting the anti-inflammatory effect and affecting the progression and development of atherosclerosis (26). Therefore, due to the complexity of the mechanism of action, some of the above studies support the link between SE and atherosclerotic plaque, perhaps through unknown underlying factors such as blood pressure, hormones, and diet. Based on the results of the MR analysis and the above conclusions, we consider it unlikely that there is a direct causal relationship between the volume of SE and CA, but we do not rule out that the volume of SE is associated with CA at other levels than the genetic level.

Coronary atherosclerotic plaque, as the main pathological feature of CHD, is closely related to the risk and outcome of various acute or recurrent coronary events (27). Although our study does not support the direct causal relationship between the volume of SE and CA, high-intensity interval training (HIIT) with a certain dose of SE has become a common exercise prescription for CR. CR is a key component of the comprehensive treatment of CHD, and its exercise prescription consists of four basic elements: exercise frequency, exercise intensity, exercise time, and exercise type. HIIT has been shown to improve exercise tolerance and vascular function in patients with cardiovascular disease, which is a method of exercise that consists of alternating between SE and short bursts of low-intensity exercise or rest at scientifically designed exercise volumes (28). Studies have shown that HIIT can delay the progression of atherosclerotic CHD and reduce the atherosclerotic volume in residual CA plaques after PCI, which may be related to the mechanism of the influence of changes in coronary blood flow on endothelial shear stress after SE. HIIT can be further classified into low- and high-volume HIIT based on the total time spent in the active interval (29). However, there is a lack of consensus on the optimal prescription of HIIT for patients with CHD participating in CR. The study by Tjønna et al. (30) randomized 26 participants into either a high-volume HIIT group (4 × 4 min) or a low-volume HIIT group (1 × 4 min) for exercise training three times a week. This 10-week study showed that the duration of low-volume HIIT was shorter than that of high-volume HIIT, and there was no significant difference in improving maximum oxygen uptake and blood pressure levels compared to large-volume HIIT training (*P* = 0.23; *P* = 0.99). However, it is regrettable that this study was conducted only in healthy individuals. A 12-week CR study of 200 patients with CHD showed that three high-volume HIIT sessions per week resulted in significant improvements in peak oxygen uptake, peripheral endothelial function, quality of life, and cardiovascular risk factors in patients with CHD (31). Another study involving 382 CHD patients also found that low-volume HIIT can improve cardiorespiratory function more effectively than moderate-intensity steady-state exercise training (32). Based on our findings, while there is no direct causal relationship between the volume of SE and CA, multiple studies support that a certain volume of SE can positively impact the heart in terms of cardiopulmonary function, blood pressure, and quality of life. Therefore, when it comes to heart rehabilitation, setting an appropriate exercise volume under scientific guidance and choosing an appropriate exercise intensity intermittent training can aid in beneficial CR training.

There are several limitations in our study: First, only a limited number of instrumental variables are significant in the GWAS of DOSE and FOSE, which is vulnerable to bias. The findings should be interpreted cautiously. Second, the heterogeneity of this study may result from the inevitable differences in the study population and sample size limitations in the original study. The GWAS data used in this study are mainly European populations, thus this conclusion cannot represent Asian or other non-European populations. Third, due to the linear effect assumption of the MR analysis, we were unable to assess the nonlinear association between these exposures and CA. Fourthly, due to the availability of data, we used two basic parameters of the volume of SE (duration and frequency of SE) to conduct MR Analysis. In the future, more large-scale research on the volume of SE is needed. Finally, the GWAS data extracted in this analysis did not include the results of stratified analysis of personal characteristics (such as age, gender, etc.) and types of intense exercise (aerobic exercise, resistance exercise, etc.), making it impossible to conduct further subgroup analysis on more specific information, and further research may be needed.

## 5 Conclusion

In conclusion, based on the results of MR obtained from large-scale GWAS pooled statistics, our two-sample MR study suggested that the causal association of duration and frequency of SE with CA was not found. This research has the potential to guide the choice of the scientific and correct volume of SE to CR.

## Data Availability

The original contributions presented in the study are included in the article/Supplementary Material. Further inquiries can be directed to the corresponding authors.
