# Supplementary material for: No causal association between the volume of strenuous exercise and coronary atherosclerosis: a two-sample Mendelian randomization study
Source: Front Cardiovasc Med. 2024 Apr 25;11:1344764. doi: 10.3389/fcvm.2024.1344764 (PMC11079240; doi:10.3389/fcvm.2024.1344764)
Supplement: Supplementary file 1 [file Table1.docx]

**Supplementary Tables**

**Supplementary Table 1:** UK Biobank touch-screen questionnaire: final version

| Unique Name | Question Stem | Responses | Validations | Actions | Hints |
| --- | --- | --- | --- | --- | --- |
| WP4C3 | How many times in the last 4 weeks did you do strenuous sports? | SELECT one of 8 from  1 :Once in the last 4 weeks  2 :2-3 times in the last 4 weeks 3 :Once a week  4 :2-3 times a week  5 :4-5 times a week  6 :Every day  -1 :Do not know  -3 :Prefer not to answer |  | Goto WP4E3 | If you are unsure, please provide an estimate or select Do not know. |
| WP4E3 | Each time you did strenuous sports, about how long did you spend doing it? | SELECT one of 9 from  1 :Less than 15 minutes  2 :Between 15 and 30 minutes  3 :Between 30 minutes and 1 hour  4 :Between 1 hour and 1½ hours 5 :Between 1½ hours and 2 hours 6 :Between 2 and 3 hours  7 : Over 3 hours  -1 :Do not know  -3 :Prefer not to answer |  | Goto WP11 | If you are unsure, please provide an estimate or select Do not know. |

**Supplementary Table 2:** Characteristics of genetic instruments associated with DOSE;

| SNP | chr | pos | A1 | A2 | eaf | beta | se | pval | F |
| --- | --- | --- | --- | --- | --- | --- | --- | --- | --- |
| rs115215870 | 3 | 15897136 | A | G | 0.020181 | 0.135608 | 0.0280855 | 1.40E-06 | 23.31244465 |
| rs116736246* | 12 | 123301358 | A | T | 0.083433 | 0.0647693 | 0.0141022 | 4.40E-06 | 21.09338035 |
| rs117068173 | 7 | 52909632 | A | C | 0.068095 | -0.0711131 | 0.0151667 | 2.70E-06 | 21.98358644 |
| rs12674653 | 8 | 142606193 | C | T | 0.649546 | -0.0384604 | 0.00825493 | 3.20E-06 | 21.70612601 |
| rs12756104* | 1 | 96810865 | A | T | 0.49681 | -0.0365667 | 0.00774284 | 2.30E-06 | 22.30244535 |
| rs12927311 | 16 | 26551874 | C | T | 0.616035 | -0.036983 | 0.00798605 | 3.60E-06 | 21.44478722 |
| rs28367980 | 1 | 21585580 | A | G | 0.00996 | 0.209975 | 0.0412558 | 3.60E-07 | 25.90279748 |
| rs717997 | 12 | 84133974 | G | A | 0.577938 | -0.0360383 | 0.00782081 | 4.10E-06 | 21.23276917 |
| rs9373403 | 6 | 144122327 | A | G | 0.341978 | 0.0385305 | 0.00811101 | 2.00E-06 | 22.56529067 |

SNP, single nucleotide polymorphism; A1, effect allele; A2, other allele; se, standard error; *: excluded SNP in final analysis.

**Supplementary Table 3:** Characteristics of genetic instruments associated with FOSE;

| SNP | chr | pos | A1 | A2 | eaf | beta | se | pval | F |
| --- | --- | --- | --- | --- | --- | --- | --- | --- | --- |
| rs11033079 | 11 | 35362050 | T | G | 0.390395 | -0.037202 | 0.0075806 | 9.20E-07 | 24.08279628 |
| rs141371509 | 5 | 55994713 | T | C | 0.020769 | 0.120313 | 0.0258705 | 3.30E-06 | 21.62703408 |
| rs144057218 | 21 | 44415351 | C | A | 0.019711 | -0.134061 | 0.0280718 | 1.80E-06 | 22.80583009 |
| rs1468033 | 17 | 78916953 | A | G | 0.576148 | -0.0354657 | 0.00755623 | 2.70E-06 | 22.02866772 |
| rs2671783 | 10 | 87051699 | T | C | 0.068996 | 0.0691584 | 0.0147016 | 2.50E-06 | 22.12800022 |
| rs76082247* | 6 | 88114755 | T | A | 0.012406 | 0.165426 | 0.0340053 | 1.10E-06 | 23.66441721 |
| rs9300279 | 12 | 128586329 | G | A | 0.709068 | -0.0417726 | 0.00850866 | 9.10E-07 | 24.10139454 |
| rs970860 | 1 | 109131023 | C | A | 0.413295 | -0.036807 | 0.00755707 | 1.10E-06 | 23.72113415 |
| rs9836806 | 3 | 115252276 | A | G | 0.945737 | -0.0793334 | 0.0164537 | 1.40E-06 | 23.24697683 |

SNP, single nucleotide polymorphism; A1, effect allele; A2, other allele; se, standard error; *: excluded SNP in final analysis.
